# Atypical sensory processing pattern following median or ulnar nerve injury — a case-control study

**DOI:** 10.1186/s12883-018-1152-y

**Published:** 2018-09-19

**Authors:** Pernilla Vikström, Anders Björkman, Ingela K. Carlsson, Anna-Karin Olsson, Birgitta Rosén

**Affiliations:** 1 Department of Translational Medicine – Hand Surgery, Skåne University Hospital and Lund University, Jan Waldenströms gata 5, SE-205 03 Malmö, Sweden; 2 Department of Psychiatry, NU Health Care, Trollhättan and Department of Psychology, Karlstad University, Karlstad, Sweden

**Keywords:** Sensory, Median nerve, Ulnar nerve, Injury, Sensory profile

## Abstract

**Background:**

Due to brain plasticity a transection of a median or ulnar nerve results in profound changes in the somatosensory areas in the brain. The permanent sensory deprivation after a peripheral nerve injury might influence the interaction between all senses.

The aim of the study was to investigate if a median and/or ulnar nerve injury gives rise to a changed sensory processing pattern. In addition we examined if age at injury, injured nerve or time since injury influence the sensory processing pattern.

**Methods:**

Fifty patients (40 men and 10 women, median age 43) operated due to a median and/or ulnar nerve injury were included. The patients completed the Adolescent/Adult Sensory Profile questionnaire, which includes a comprehensive characterization on how sensory information is processed and how an individual responds to multiple sensory modalities. AASP categorizes the results into four possible Quadrants of behavioral profiles (Q1-low registration, Q2-sensory seeking, Q3-sensory sensitivity and Q4-sensory avoiding). The results were compared to 209 healthy age and gender matched controls. Anova Matched Design was used for evaluation of differences between the patient group and the control group. Atypical sensory processing behavior was determined in relation to the normative distribution of the control group.

**Results:**

Significant difference was seen in Q1, low registration. 40% in the patient group scored atypically in this Quadrant compared to 16% of the controls. No correlation between atypical sensory processing pattern and age or time since injury was seen.

**Conclusion:**

A peripheral nerve injury entails altered sensory processing pattern with increased proportion of patients with low registration to sensory stimulus overall. Our results can guide us into more client centered rehabilitation strategies.

## Background

An injury to a major nerve in the upper extremity in adults causes long-lasting disability due to loss of fine sensory and motor function [1, 2]. In addition, such injuries often cause psychological stress and may have devastating long-term effects on ADL and quality of life [3–9].

Sensibility is the function most seriously affected by a nerve injury [4, 10]. However, “sensibility” is much more than just touch sensation. Touch is one aspect of perception where perception is a range of processes involved in turning sensations from all sense organs into meaningful information [11]. These perceptual processes are necessary to help us, among other things, orient in the environment and sort out the importance from all stimulus we are exposed to continuously, including touch stimulus.

The interaction between all senses are crucial for our understanding of, and interaction with, the surrounding world, where cross-modal association areas of the brain merge stimulus from all senses [12, 13].

A person’s *“sensory profile”* describes how an individual processes information from all senses and the sensory profile is thus highly individual. The Adolescent/Adult Sensory Profile (AASP) is a standardized self-reported questionnaire that classifies sensory experiences and behavioral responses [14]. It includes a comprehensive characterization on how sensory information is processed and how an individual responds to multiple sensory modalities (Touch, Taste/Smell, Visual, Auditory, Movement and Activity). Based on the results from the questionnaire, a sensory profile is created [15, 16]. The concept was developed from Dunn’s model of Sensory Processing. Dunn proposes that four sensory processing patterns characterize the perceptual process. These patterns are thought to arise from both individual differences in neurological thresholds to notice or react to stimuli, but also from self-regulation strategies, the so-called response behavior. The neurological thresholds refers to how readily the nervous system detects and reacts to stimuli, a lower threshold, the greater the probability of nervous system will be to detect and react to stimuli. The self-regulatory behavioral responses depicts how people behave in responses to stimuli according to their neurological thresholds. A person can respond in accordance to the neurological threshold (passive behavior response) or by counteract their neurological threshold (active behavior response). Combinations of these dimensions gives us four sensory processing styles [17]. AASP has primarily been used in rehabilitation of neuropsychiatric disorders and is suggested to be useful in planning clinical interventions [14]. In addition, AASP has also been shown to facilitate planning of clinical interventions in a few studies of patients with physical disorders such as atopic dermatitis, stroke and asthma [18–22].

A transection of a median or ulnar nerve results in rapid and profound changes in the somatosensory areas in the brain due to brain plasticity [23–28], and there are changes in the peripheral nerve as well [29]. Therefore, there are reasons to believe that the permanent sensory deprivation after a peripheral nerve transection might interact and influence the cross-modality between all senses as well as the higher cognitive functions in the brain. The proportionally large representation of the hand in the somatosensory cortex [30] is another reason to believe that a person’s sensory profile may change. This depends on whether the extensive changes in this area can contribute to changes in other areas after median and/or ulnar nerve injury. Hypothetically, the changes in the somatosensory cortex may also affect processing of information from other senses.

If sensory processing changes could be demonstrated, it would teach us more about the plasticity of the brain following a peripheral nerve transection. In addition, knowledge about the patient’s sensory profile might improve the possibility to individualize rehabilitation following nerve transection. Hence, given that an adult person with a severe nerve injury has a permanent limited perception of touch we hypothesized that patients with such injuries have an atypical experience and behavior in sensory processing overall.

Age at injury is a well-known influencing factor for sensory outcome after a nerve injury [31, 32] and improvement of sensory function following a major nerve trauma continues for years [33–35].

The aim of the present study was to investigate if a median and/or ulnar nerve transection gives rise to a changed sensory profile. An additional aim was to investigate how age at injury and time since injury influence the sensory profile.

## Methods

### Participants

Fifty adult (> 18 years) patients with at least 50% repair of the median and/or ulnar nerve were included in the study. All available patients from two earlier studies were asked to participate in the present study. The inclusion criteria were described in detail in those reports [36, 37]. Exclusion criteria were severe psychiatric or neurological disorder and communication problems due to language difficulties. Each patient was matched with four to six individual age (± 2 years) and gender matched controls from a healthy control group. The matched controls were extracted from the normative population from validation of the Sensory Profile into Swedish [38]. Depending on the varying amount of available matched controls in the normative population the number of controls vary between four and six.

### Measures

The adolescent/Adult Sensory Profile [14] is a self-reported 60 item questionnaire based on Dunn’s Model of Sensory Processing [17].

The 60 items are divided into four quadrants, based on a combination of behavioral response and neurological threshold. The four Quadrants are Low registration, Sensation Seeking, Sensory Sensitivity, and Sensory Avoiding. The questions concern experiences of sensory processing in everyday sensory experiences across different sensory processing domains (Taste/Smell, Movement, Visual, Touch, Activity and Auditory). Every item is scored on a 5-point scale where 1 = almost never, to 5 = almost always. The sum of the scores for each Quadrant and the six Domains are calculated from the answers.

### Data analysis

To investigate whether a median and/or ulnar nerve injury gives rise to a changed sensory profile, Anova Matched Design was used for calculation of differences between the patient group and the age and gender matched control group. This was done for each of the four Quadrants (Q1-low registration, Q2-sensory seeking, Q3-sensory sensitivity and Q4-sensory avoiding) and the six Domains (Taste/Smell, Movement, Visual, Touch, Activity and Auditory). Level of significance was ≤0.05.

For the Quadrants/Domains with a significant difference between the groups, the percentage of patients who scored Atypically High and Atypically Low was calculated. A patient score ± 1 SD of the control group mean was categorized as “Atypically High” or “Atypically Low” respectively. We compared this percentage against a test value of 16%, indicating ± ≥ 1 SD, from the control group mean, which is interpreted as “more/less than most people”.

The same calculation was then made for the scores of Definitely High/Low. A patient score ± 2 SD of the control group mean was categorized as “Definitely High” or “Definitely Low” respectively. We compared this percentage against a test value of 2.5%, indicating ± ≥ 2 SD from the control group mean, which is interpreted as “much more/much less than most people” [39]. Deviations from the mean were analyzed separately for each direction and confidence interval was calculated according to Binomial distribution.

Spearman correlation was used to examine the relationship between the Sensory Profile™ score and the possible influencing factors age and time since injury.

### Ethics

The study was approved by the Ethical Committee of Lund University and it was conducted.

according to the declaration of Helsinki. All participants gave written consent.

## Results

### Participants

Fifty patients were included in the study. One patient was lost due to incomplete filling of the AASP questionnaire (Table 1).

**Table 1:** Demographics

	Patient group *n* = 49	Control group *n* = 209
Gender Men/Women	39/10	164/45
Age^a^	43 (19–75)	44 (18–76)
Months since injury^a^	24 (7–108)	–
Injured nerve median/ulnar/both	18/27/4	–
Complete/Partial nerve transection	45/4	–

^a^Presented with median (range)

### Sensory profile quadrants comparison

**Fig. 1:**
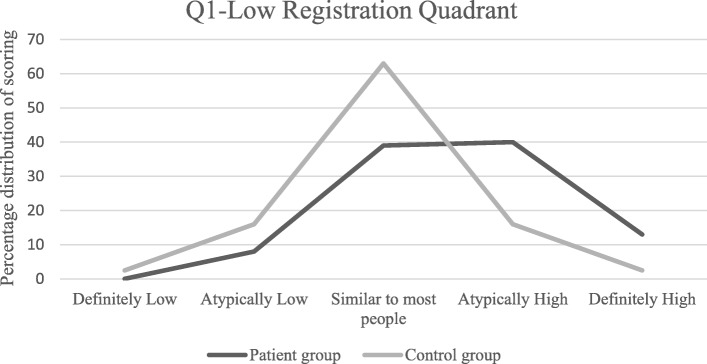
Distribution of patient group Sensory Profile™ score in the Low Registration Quadrant in comparison to the normative distribution curve of the control group

When comparing patients with controls, the results in the Low Registration Quadrant differed significantly (Table 2). An increased amount of the patients scored Atypically/Definitely High in the Low Registration Quadrant and an increased amount of patients scored Atypically/Definitely Low in the Low Registration Quadrant compared to their controls (Table 3 and Fig. 1). No significant differences were seen for the other three Quadrants.

**Table 2:** Significance of differences between patients and controls

	*p*-value
Low registration, Q 1	0.029
Sensory seeking, Q 2	0.956
Sensory sensitivity, Q 3	0.206
Sensory avoiding, Q 4	0.268

Level of significance = *p*-value ≤0.05

**Table 3:** Percentage distribution of scoring in the Low Registration Quadrant

	Patient group	95% CI	Control group
Atypically High	40%	32–48	16%
Definitely High	13%	8–19	2.5%
Atypically Low	8%	4–14	16%
Definitely Low	0%	0–4	2.5%

Percentage distribution of scoring Atypically High/Low (± 1 SD compared with the control group mean) and Definitely High/Low in Q1-Low Registration Quadrant (± 2 SD compared with the control group mean)

### Sensory profile domains comparison

No differences between the patients and the controls were seen in any of the six specific Domains (Taste/Smell, Movement, Vision, Touch, Activity and Auditory).

### Influencing factors

No statistical correlations were seen between Sensory Profile™ score and age or time since injury.

## Discussion

This study shows that patients with median or ulnar nerve injuries have an altered sensory processing pattern with increased incidence of low registration of impressions from all senses.

Low registration means high neurological thresholds, which in turn means that these persons fail to detect stimuli that others notice and more intense stimuli are needed for the nervous system to respond and enable the patients to sustain attention [39]. Atypical sensory processing patterns have also been seen in adults with atopic dermatitis and following a stroke, but in contrast to nerve injured patients, the patients with atopic dermatitis and post stroke showed decreased neurological thresholds, which in turn means that they respond to a low amount of stimulus [19, 22]. The present study supports the idea that the sensory profile also changes in physical disorders. It is important to remember that a multitude of combinations in scores in the four Quadrants are possible, which gives the individual a unique sensory processing pattern. The score does not tell when a pattern is problematic for the individual in daily life, instead it shows how the person compares to a larger matched control group [39] and gives insight into personal behavior and responses to different environments [14]. Problems arise only when there is a conflict between the patient’s will or wishes and the current performance [39], meaning that there are no definitive cut-off scores when the profile is problematic. We have not investigated the relationship between divergent sensory processing pattern and quality of life. This, on the other hand, has been investigated in a very recent study on patients with multiple sclerosis where a significant correlation was found between high scores in Low Registration Quadrant and reduced quality of life [40]. In addition, Kinnealy [41] also demonstrated a correlation between Low Registration Quadrant and reduced quality of life including all four areas encompassing emotional health in Short Form-36 Health Survey.

Sensory relearning is a vital part of the rehabilitation following peripheral nerve repair and it is the training technique used to prepare and “teach” the somatosensory cortex to interpret the new afferent signaling at touch [1].This training is a process of stimulating the brain through the use of cognitive learning techniques. Sensory relearning is designed to stimulate sensory areas in order to improve the cortical processing of the changed afferent input [35] and starts immediately after the nerve repair [1]. Sensory relearning uses the plastic capacity of the brain for therapeutic purpose, i.e. guided plasticity [42] and also the cross-modal capacity of the brain [13]. By using different techniques for guided plasticity, such as motor or sensory observation [43, 44] and motor or sensory imagery exercises [45, 46], activation of the somatosensory cortex is achieved. These techniques are used in rehabilitation during the sensory deprivation after the nerve injury to stimulate the somatosensory cortex in in the initial phase after the nerve transection, before the axons have reinnervated their targets in the hand. Furthermore, to replace one sensory modality with another, cross-modal plasticity, is a concept that in previous studies has been proved beneficial for sensory re-learning after peripheral nerve injuries [9, 36, 47–50].

The results from the current study may support that cross-modal rehabilitation techniques, with multiple simultaneous sensory stimulus, would be beneficial in sensory relearning since it is suggested for people scoring atypically high in the Low Registration Quadrant, to increase the intensity of stimuli [39]. The literature about sensory profile also advocates for such “low registrators” to vary the kind of stimulus and to slow down the pace of presentation of stimuli, with purpose to let the patient get enough time to detect and process the information.

In addition, knowledge about sensory profile can help to individualize components in the rehab design such as the amount and frequency of training sessions and visits to the hospital. The individual patient can then practice, understand and exercise at his/her own pace. This is in line with what was reported in a qualitative report of patients’ experiences of early sensory relearning where it was found that there is a great variation in the need of guidance in the specific training –sensory relearning – following a nerve injury [37]. An advantage for people who score high in the Low Registration Quadrant in the Sensory Profile questionnaire is that they find it easier to focus on tasks that they find interesting, even in distracting environments [39]. Ideally this should be used in sensory relearning however, the challenge is to make the relearning interesting and meaningful for the patient.

There are limitations in this study. The number of individuals is limited, but a strength is that we had access to a large control group where every patient could be matched to four to six individual controls. A confounding factor in this study is the possibility of influencing neuropsychological factors that are not investigated here, but which contribute to the result in the Sensory Profile. The AASP questionnaire was developed for use in neuropsychiatric disorders. However, several studies including patients with more somatic disorders such as atopic dermatitis, stroke and multiple sclerosis [19, 22, 40] have revealed interesting findings in sensory processing when using the AASP. Our findings can also guide the rehabilitation strategies. This exploratory study also gives us an idea of the deviation and atypical sensory processing patterns that exist in patients with median/ulnar nerve transection.

Surprisingly, neither age nor time since injury influenced the sensory processing patterns. An addition of direct measures of the patient’s sensory function [51] maybe had gained knowledge of if there is a relationship between abnormal sensory processing patterns and sensory outcome following peripheral nerve injury.

Previous research on patients with severe peripheral nerve injuries [37] showed that a majority of patients expressed a need for strong support from the therapist. Furthermore, at least 14% of the patients expressed a need for creating routines for their sensory relearning program. The present study show that the patients with severe peripheral nerve injuries have an atypically sensory processing pattern. Proposed interventions for these individuals with atypical high scoring in the Low Registration Quadrant [39] e.g. intense, ideally multisensory, stimuli and to give the person enough time to perceive the stimuli. Multisensory stimulations in sensory re-learning have also been suggested previously [52] and that is also in line with our results here. In order to meet the needs found in this study, an example of a multisensory technique that can be used more in client centered rehabilitation is in a familiar self-chosen meal situation. In the meal situation the patient should be encouraged to use all senses. For example, when handling an orange, “sense not only the texture and shape but also the scent, color and taste”. In such an everyday situation the amount of sensory input is increased, which may be useful in rehabilitation for these “low registrators”.

Among proposed interventions for patients scoring high in Low Registration Quadrant [39] are also repeated oral and written information, as well as individually structured time and environments with varying and contrasting stimulus.

Future studies should focus on the development of multisensory rehabilitation applications with enhanced opportunity for repeated information, a motivational client centered approach in training and variation of the intensity and complexity of stimuli/objects in sensory relearning.

## Conclusion

We have here showed that a peripheral nerve injury causes altered sensory processing pattern with an increased proportion of patients with low registration to sensory stimulus overall, and the results can guide us into more client centered rehabilitation strategies.

## Abbreviations

AASP: Adolescent/Adult Sensory Profile
ADL: Activities of Daily Life
